# Establishment and validation of individualized clinical prognostic markers for LUAD patients based on autophagy-related genes

**DOI:** 10.18632/aging.204097

**Published:** 2022-09-29

**Authors:** Yuchang Fei, Junyi Xu, Liping Ge, Luting Chen, Huan Yu, Lei Pan, Peifeng Chen

**Affiliations:** 1 Department of Integrated Chinese and Western Medicine, The First People’s Hospital of Jiashan, Jiaxing, Zhejiang, China; 2 Information Center, The First People’s Hospital of Jiashan, Jiaxing, Zhejiang, China; 3 Department of Breast Surgery, Fudan University Shanghai Cancer Center, Xuhui, Shanghai, China; 4 Department of Integrated Chinese and Western Medicine, The First People’s Hospital of Wenling, Taizhou, Zhejiang, China; 5 Ningbo Yinzhou Second Hospital, Ningbo, Zhejiang, China; 6 Department of Oncology, The First Affiliated Hospital of Zhejiang Chinese Medical University, Hangzhou, Zhejiang, China

**Keywords:** lung adenocarcinoma, autophagy, prognosis, bioinformatics, biomarkers

## Abstract

There is considerable heterogeneity in the genomic drivers of lung adenocarcinoma, which has a dismal prognosis. Bioinformatics analysis was performed on lung adenocarcinoma (LUAD) datasets to establish a multi-autophagy gene model to predict patient prognosis. LUAD data were downloaded from The Cancer Genome Atlas (TCGA) database as a training set to construct a LUAD prognostic model. According to the risk score, a Kaplan-Meier cumulative curve was plotted to evaluate the prognostic value. Furthermore, a nomogram was established to predict the three-year and five-year survival of patients with LUAD based on their prognostic characteristics. Two genes (ITGB1 and EIF2AK3) were identified in the autophagy-related prognostic model, and the multivariate Cox proportional risk model showed that risk score was an independent predictor of prognosis in LUAD patients (HR=3.3, 95%CI= 2.3 to 4.6, *P*< 0.0001). The Kaplan-Meier cumulative curve showed that low-risk patients had significantly better overall (*P*<0.0001). The validation dataset GSE68465 further confirmed the nomogram’s robust ability to assess the prognosis of LUAD patients. A prognosis model of autophagy-related genes based on a LUAD dataset was constructed and exhibited diagnostic value in the prognosis of LUAD patients. Moreover, real-time qPCR confirmed the expression patterns of EIF2AK3 and ITGB1 in LUAD cell lines. Two key autophagy-related genes have been suggested as prognostic markers for lung adenocarcinoma.

## INTRODUCTION

Lung cancer is one of the leading causes of cancer death worldwide, causing approximately 1.76 million deaths annually [1]. Among major histological types, lung adenocarcinoma has gradually increased in incidence in most countries during the past few decades and has become the most common histological type, accounting for approximately 40% of all histological types [2]. However, there is considerable heterogeneity in the genomic drivers of lung adenocarcinoma, and the prognosis is not optimistic. 75% of patients are often diagnosed at an advanced stage, and the average five-year survival rate is less than 15% [3–5]. Although many potential therapeutic targets have been identified in LUAD, currently identified mutation genes are not detected in most LUAD patients. Based on this, more effective biomarkers are needed to better detect, diagnose and evaluate the prognosis of LUAD.

Autophagy is a degradation process in which cells recover damaged organelles and remove excess damaged organelles through lysosomes to maintain homeostasis [6]. It is widely found in eukaryotic cells as a way of cell self-renewal. Autophagy belongs to type II programmed cell death and plays a wide range of pathophysiological roles in the occurrence and development of multiple diseases, such as neurodegenerative diseases, cancer, and autoimmune diseases [7, 8]. Nevertheless, autophagy plays a dual role in promoting and inhibiting tumor progression [9–11]. On the one hand, autophagy prevents cell damage and inflammation and enforces quality control of proteins and organelles during the early stages of tumor development, thus preventing tumor proliferation and invasion [12–14]. On the other hand, autophagy protects tumor cells and maintains tumor metabolism and survival as a defense mechanism in the advanced tumor stage. Such a mechanism further promotes tumor occurrence and even promotes metastasis to increase the aggressivity of cancer and eventually lead to resistance to therapeutic drugs [15–18].

To explore LUAD-related autophagy genes, we constructed LUAD-related autophagy prognostic signatures based on the LUAD dataset from The Cancer Genome Atlas (TCGA) Database. The mRNA differential expression in the TCGA-LUAD cohort was analyzed. Then we analyzed autophagy-related genes (ARGs) by KEGG [19, 20] and GO pathway analysis and constructed a protein-protein interaction (PPI) network. The Univariate Cox proportional hazards model screened 210 autophagy-related genes, further included in the Least Absolute Shrinkage and Selection Operator (LASSO) for analysis. The protein expression level of related genes was further validated in The Human Protein Atlas (HPA). A Multivariate Cox proportional-hazards model was utilized to further screen the above genes, and the prognosis model was constructed according to the clinical characteristics of the TCGA-LUAD cohort. Survival analysis was performed on the TCGA-LUAD cohort to assess the prognostic value of the risk score (the median risk score was used as the basis for grouping into High and Low-Risk groups.). We further classified the TCGA-LUAD dataset according to high and low risk and performed pathway enrichment analysis with GSEA (Gene Set Enrichment Analysis). Based on the clinicopathological characteristics of the TCGA-LUAD cohort, a nomogram was constructed to predict the three-year and five-year individual survival probability, which was further validated in the GSE68465 dataset.

## RESULTS

### Research flow chart of this study

**Figure 1:**
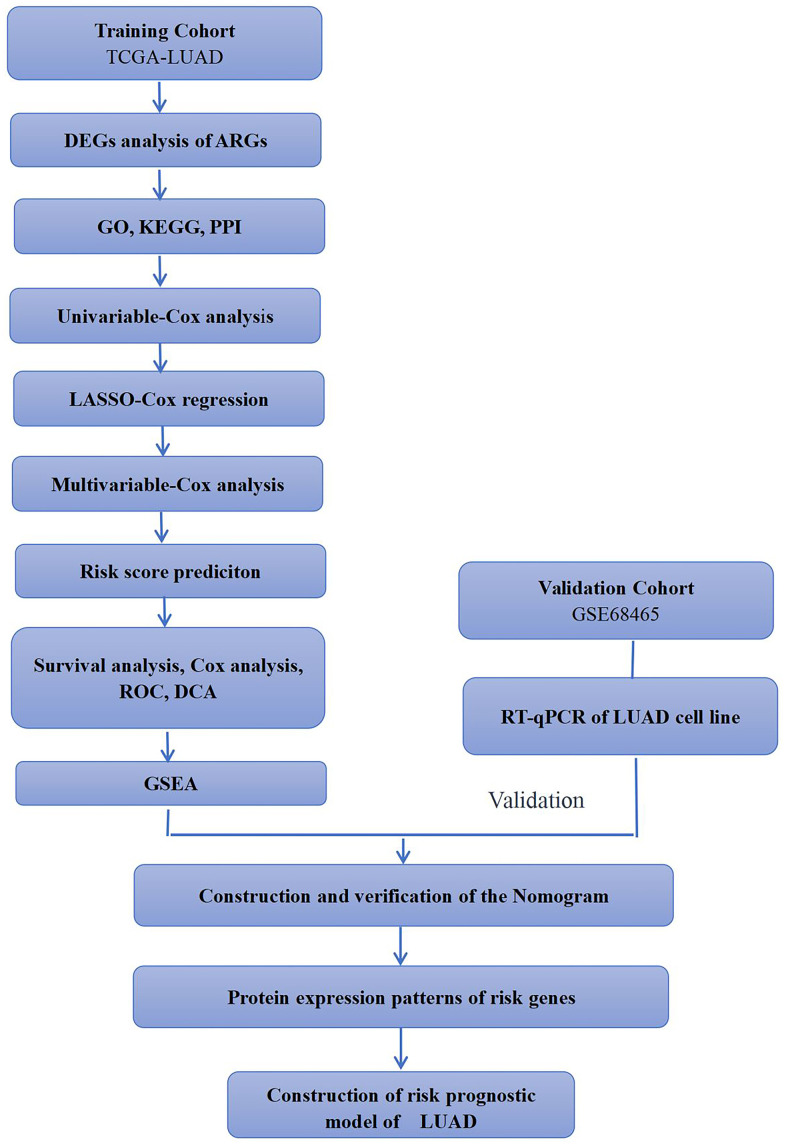
**Simple flow chart of this study.** TCGA-LUAD and GSE68465 cohorts were used for analysis in this study. Training cohorts were used to detect prognostic genes. Lasso regression model was used to establish prognostic signatures based on prognostic genes. We then confirmed the expression patterns of EIF2AK3 and ITGB1 in LUAD cell lines.

This study aimed to build a robust and reliable LUAD-ARGs risk model and conduct a downstream analysis. The research flowchart was shown in Figure 1. Differentially expressed ARGs were screened from the TCGA-LUAD dataset. Subsequently, these DE-ARGs were used to establish a specific risk model in the training dataset, which was further confirmed using the validation dataset.

### Differential expression of ARGs in LUAD

**Figure 2:**
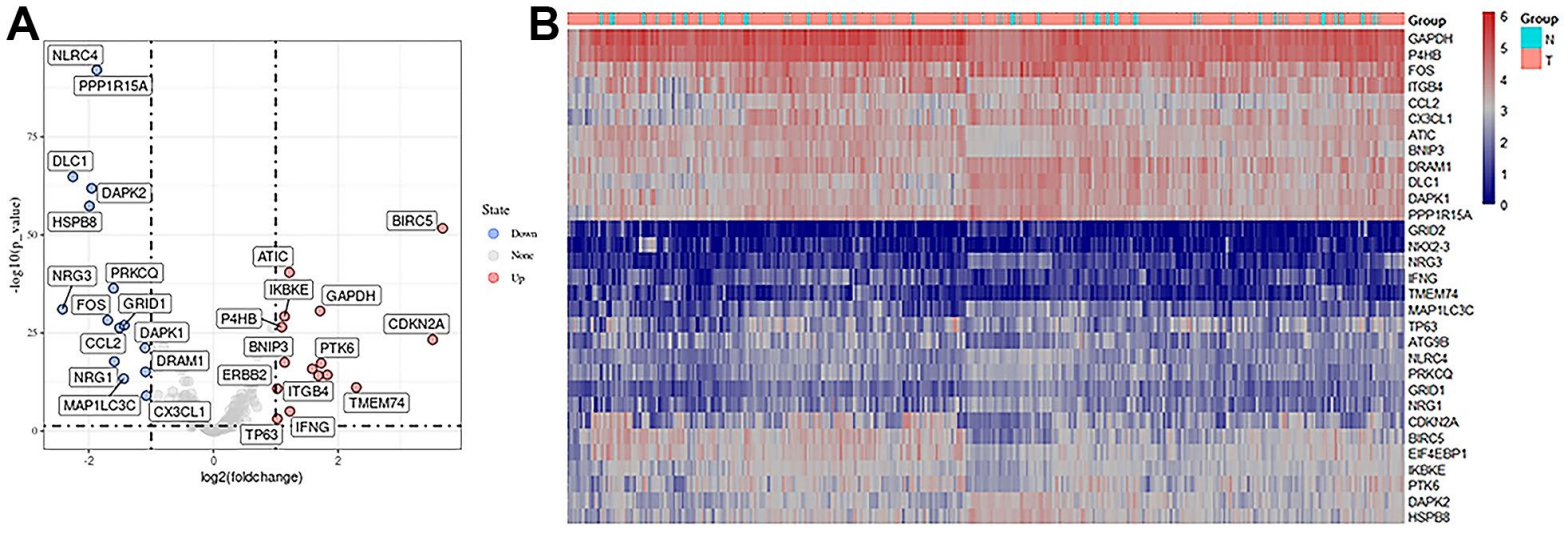
**Expression of autophagy-related differential genes.** (**A**) Volcano plot of differentially expressed autophagy-related genes, the horizontal axis was the differential expression multiple (Log2FC>2), the longitudinal axis was -log10(FDR), the blue point was the up-regulated gene, and the red point was the down-regulated gene. (**B**) Heatmaps of autophagy-related differentially expressed genes, a sample of the horizontal axis and vertical axis for different genes, red for the highly expressed genes, blue for low expressed genes (screening condition:| Log2FC | > 1, adj. p < 0.05).

There were 594 cases in the TCGA-LUAD cohort, including 535 LUAD tissue samples and 59 normal samples. Cases with missing information were removed, and a total of 518 patients with LUAD were eventually included. The corresponding clinicopathological characteristics of the TCGA-LUAD cohort were displayed in Table 1. After comparison with 232 autophagy genes in the HADb database, 210 lung adenocarcinoma-related autophagy genes were obtained. Further differentiation analysis was conducted by the R package “limma”, and screening criteria were set as follows:|Log2FC | >1, adj. *P* < 0.05. Finally, 31 ARGs were obtained, of which 12 gene expressions were upregulated, and 19 were downregulated in tumor tissues. Differential expression of these 31 genes is shown in the volcano map and heatmap (Figure 2).

**Table 1:** Clinicopathological parameters of LUAD patients in the TCGA database.

**Clinical parameters**	**Variable**	**Total(518)**	**Percentages(%)**
**Age**	<=65	251	48.5%
	>65	257	49.6%
	NA	10	1.9%
**Gender**	Female	277	53.5%
	Male	241	46.5%
**Pathological stage**	Stage I	288	55.6%
	Stage II	120	23.2%
	Stage III	78	15.1%
	Stage IV	24	4.6%
	NA	8	1.5%
**T**	Tx	3	0.6%
	T1	174	33.6%
	T2	277	53.5%
	T3	47	9.1%
	T4	17	3.3%
**N**	Tx	14	2.7%
	N0	342	66.0%
	N1	92	17.8%
	N2	67	12.9%
	N3	2	0.4%
	NA	1	0.2%
**M**	Mx	142	27.4%
	M1	23	4.4%
	M0	347	67.0%
	NA	6	1.2%
**Survival status**	Dead	183	35.3%
	Alive	335	64.7%

T, Tumour; N, Lymph Node; M, Metastasis.

### Functional enrichment analysis and PPI network of differentially expressed LUAD-ARGs

**Figure 3:**
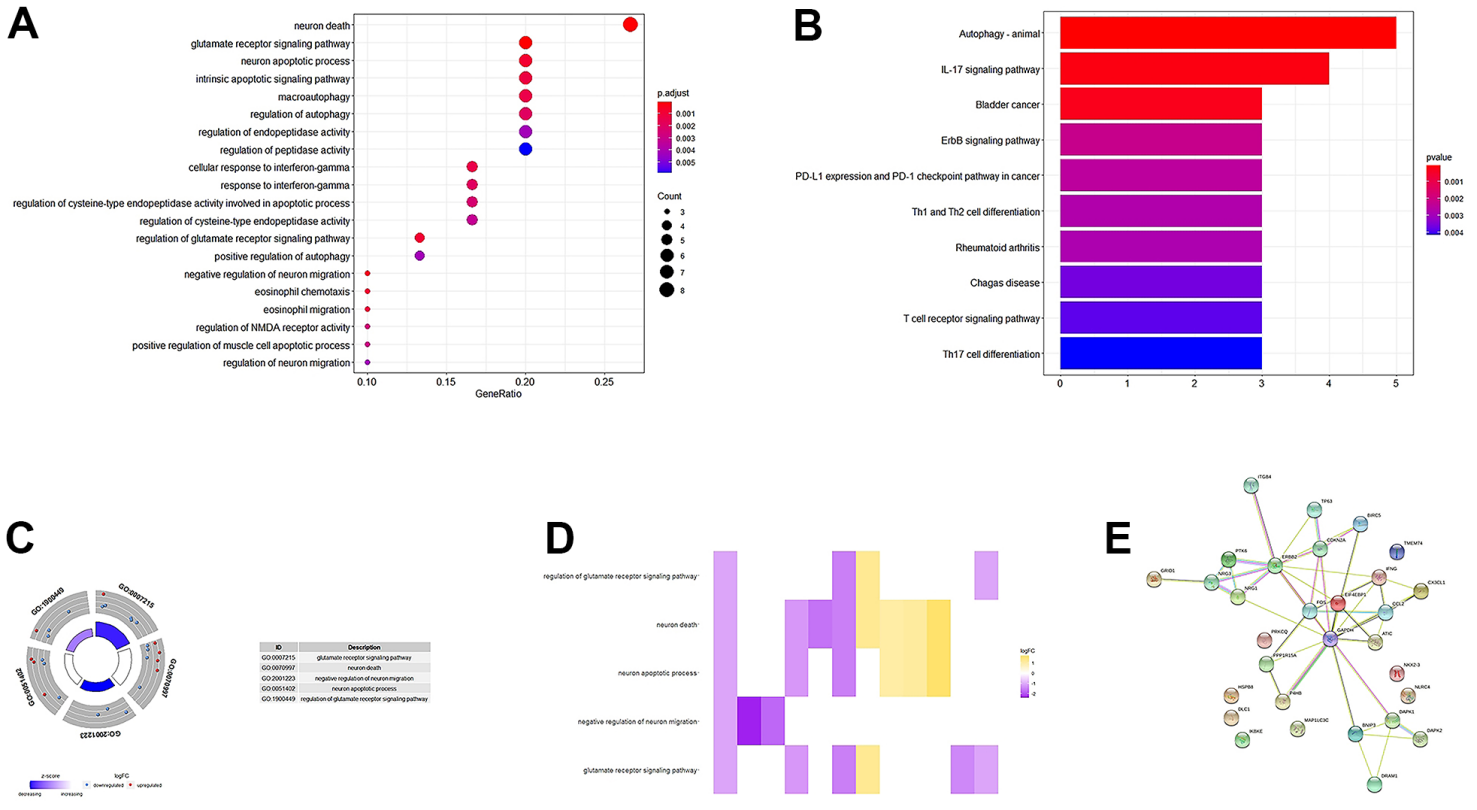
**GO, KEGG enrichment analysis, and PPI network.** (**A**, **B**) GO analysis of 31 differentially expressed autophagy-related genes. (**C**) A circle graph of the top five GO terms with the most gene abundance. (**D**) Heatmaps of the correlations between ARGs and pathways. The color of each block depends on the logFC value. (**E**) PPI network inner mapping of 31 autophagy-related differentially expressed genes.

Figure 3 summarizes the GO term and KEGG pathway enrichment analysis results for these 31 ARGs. Mainly enriched biological processes (BP) include normal death, glutamate receptor signaling pathway, neuronal apoptosis process, and apoptosis intrinsic signaling pathway (Figure 3A). Moreover, we found that autophagy, the ErbB signaling pathway, IL-17 signaling pathway, PD-L1 expression in tumors, and PD-1 checkpoint signaling pathway were enriched in these 31 genes (Figure 3B). In addition, these genes were significantly associated with negative regulation of neuron migration and neuron death during KEGG pathway enrichment analysis (Figure 3C, 3D). We constructed a PPI network of the above ARGs in String (http://string-db.org/) (Figure 3E).

### Establishment of prognostic markers and risk model for TCGA lung adenocarcinoma

**Figure 4:**
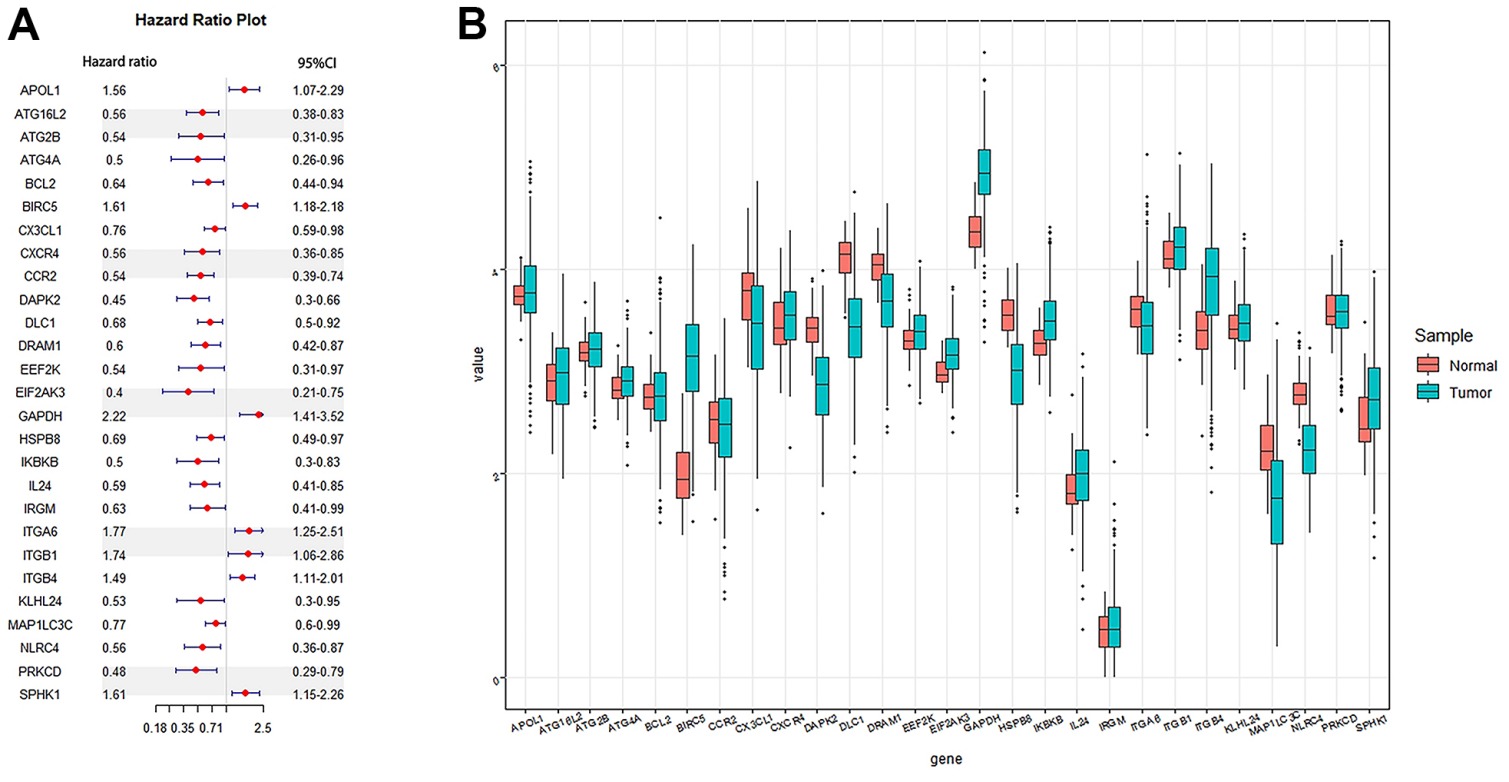
**Cox regression analysis was used to screen autophagy genes related to the prognosis of lung adenocarcinoma.** (**A**) Forest plots of autophagy genes associated with LUAD survival were screened by univariate Cox risk regression analysis (P <0.01). (**B**) Boxplot of autophagy genes associated with LUAD survival. Sample: Normal and Tumor.

**Figure 5:**
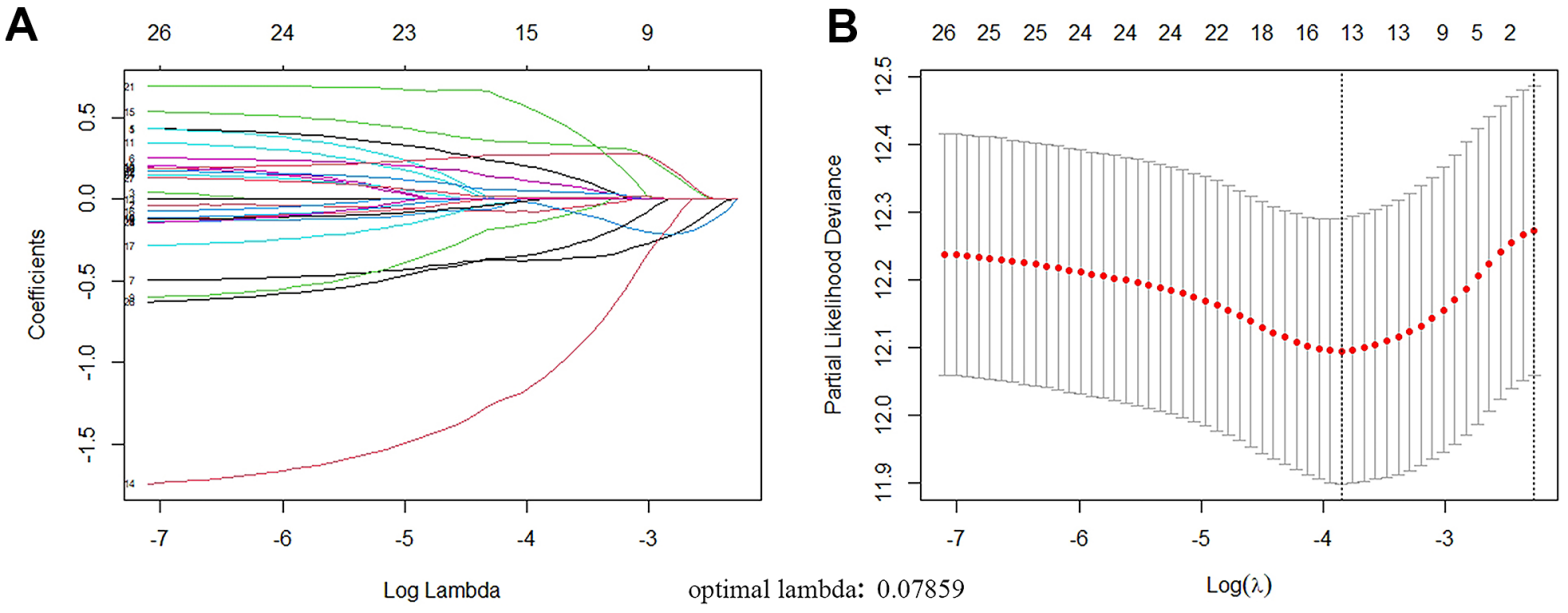
**ARGs with prognostic potential were screened by LASSO regression.** (**A**) Filter the optimal parameter (lambda) when drawing A vertical line. (**B**) The lasso coefficient distribution of 13 ARGs with non-zero coefficients was determined by the optimal lambda(0.07859).

The Univariate Cox proportional-hazards model was implemented on 210 ARGs, and 27 genes were significantly associated with TCGA-LUAD (*P*<0.01, Figure 4). Then, the 27 genes were included in the LASSO Cox analysis to remove ARGs that might be highly correlated with other ARGs (Figure 5). According to the lambda values of different genes in Lasso Cox analysis, the optimal number was 13, the optimal lambda was 0.07859. Then, the multivariate Cox proportional hazards model was utilized to further analyze the above genes. We ultimately identified two genes (EIF2AK3, ITGB1) that were related to LUAD prognosis.

**Figure 6:**
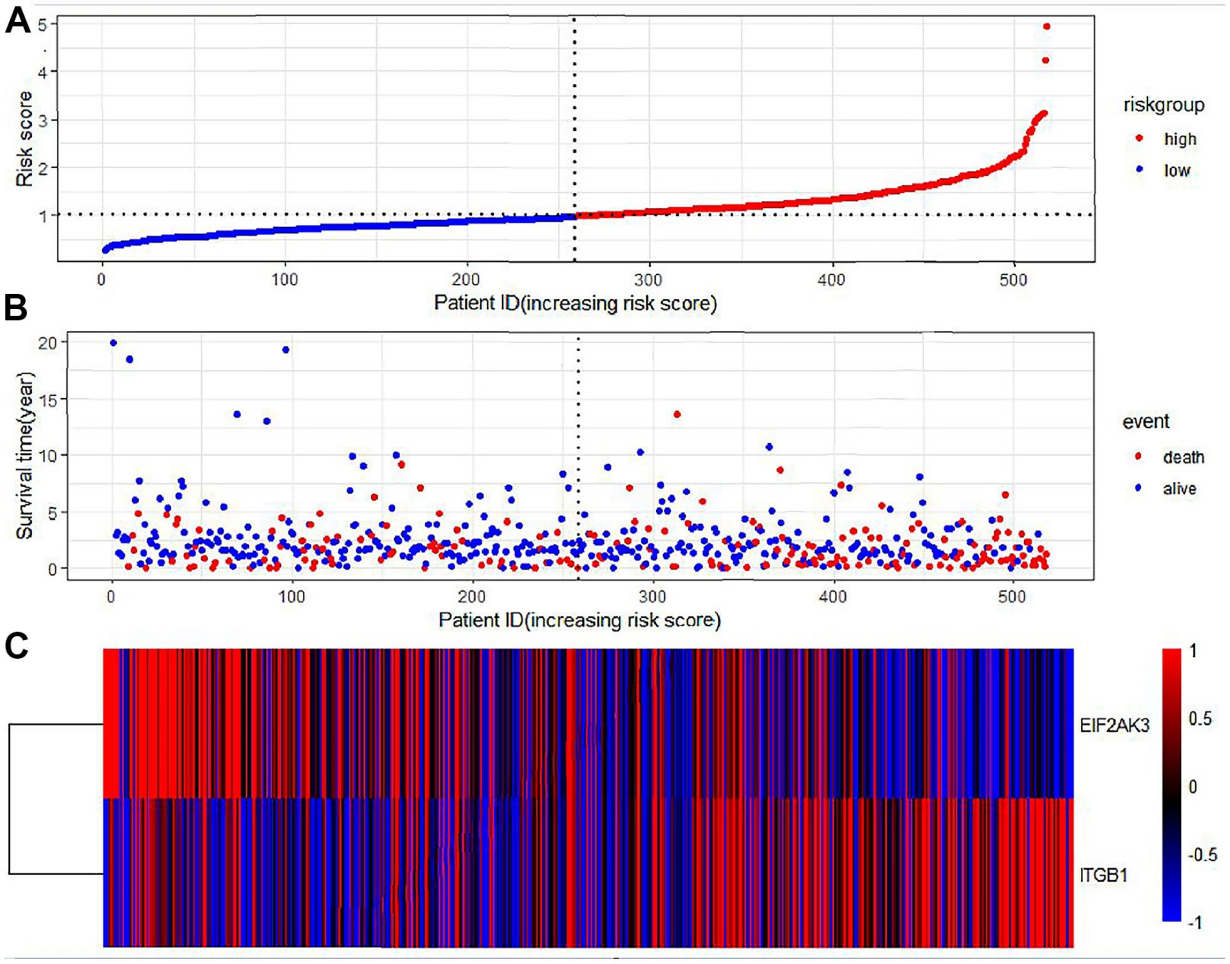
**Prognostic characteristics of autophagy genes in patients with lung adenocarcinoma.** (**A**) Distribution of risk scores in the TCGA-LUAD cohort with different risks (low: blue, high: red). (**B**) The dot plot showed the survival time and risk score in the TCGA-LUAD cohort. (**C**) Heatmap of autophagy-related gene expression profiles in LUAD prognostic characteristics.

We calculated each gene’s risk score according to ARG mRNA expression level and the regression coefficient obtained from the LASSO Cox regression analysis. The TCGA-LUAD cohort was divided into High-Risk and Low-Risk groups according to the median risk score. Figure 6 shows the distribution of risk scores in LUAD patients and their relationship with survival time. Gene expression of LUAD in the High Risk and Low-Risk groups was shown as a heatmap. The HR>1 gene (ITGB1) was considered a prognostic risk gene for LUAD, and the HR<1 gene (EIF2AK3) was considered a prognostic protective gene for LUAD (Table 2).

**Table 2:** Genes included in prognostic gene signature.

**Gene symbol**	**Full name**	**Coefficient**	**HR**	**P value**
EIF2AK3	Eukaryotic translation initiation factor 2-alpha kinase 3	-1.0834879	-3.4	0.0008
ITGB1	Integrin, beta 1	0.51830575	2.15	0.031

HR, hazard ratio.

### Autophagy-related genes as an independent prognostic factor for LUAD

**Figure 7:**
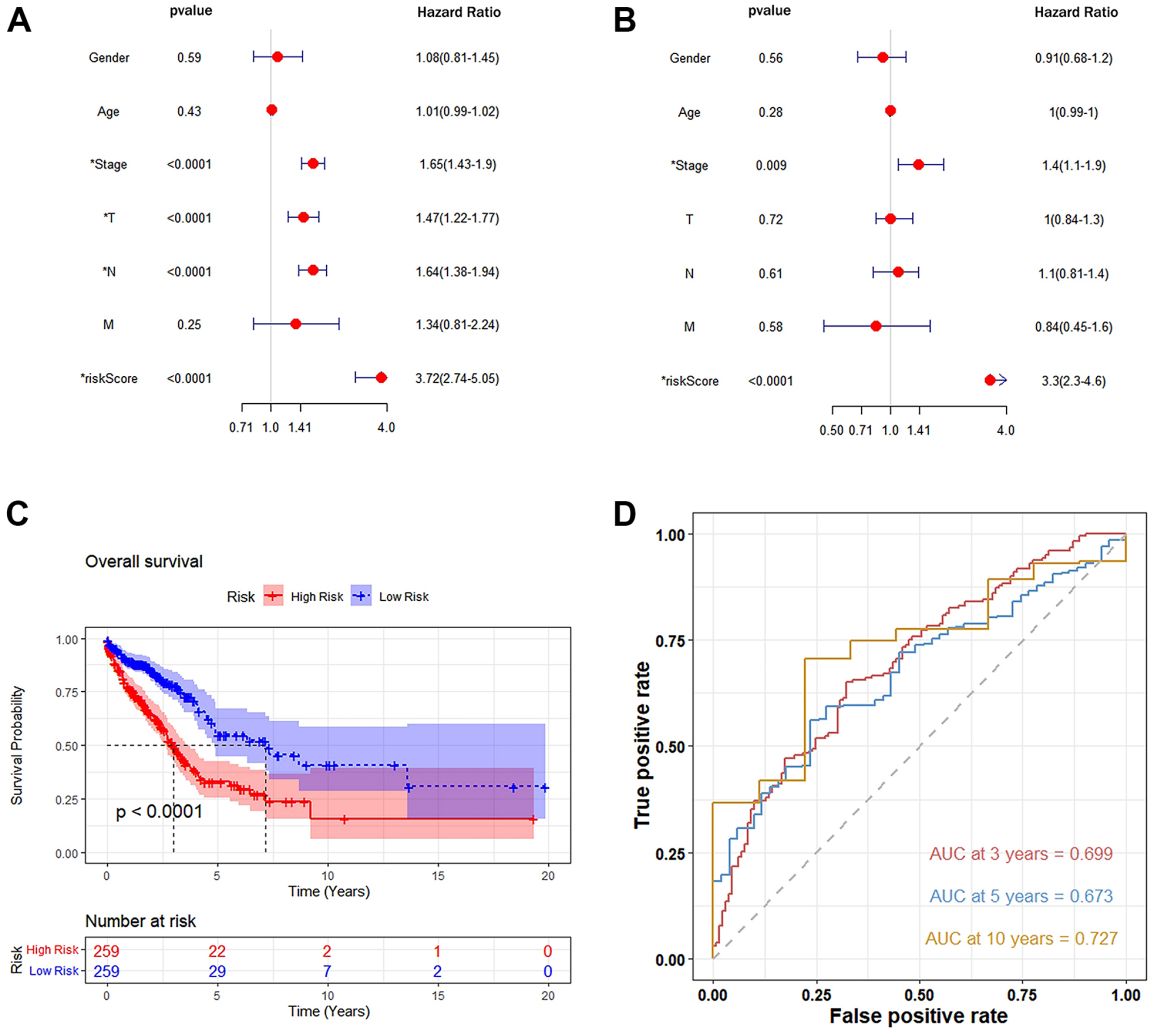
**Autophagy-related genetic markers were significantly associated with survival of lung adenocarcinoma.** (**A**) Univariate Cox risk regression analysis: forest plot of the association between risk factors and LUAD survival. (**B**) Multivariate Cox risk regression analysis: autophagy-related gene markers were independent predictors of TCGA-LUAD. (**C**) Kaplan-Meier analysis of the TCGA-LUAD cohort was significantly stratified by median risk. A high-risk score was significantly associated with poor survival in the TCGA-LUAD cohort. (**D**) Accuracy of the Time-Roc curve in predicting 3-year, 5-year, and 10-year survival in the TCGA-LUAD cohort.

The prognostic value of each gene’s risk score was calculated. In the TCGA-LUAD cohort, the univariate Cox proportional hazards model showed a remarkable correlation between risk score and overall survival (HR=3.72, 95%CI=2.74-5.05, *P*<0.0001) (Figure 7A). The Multivariate Cox proportional-hazards model showed that risk score was an independent prognostic marker for LUAD (HR =3.30, 95%CI = 2.3-4.6, *P*< 0.0001) (Figure 7B). Moreover, the Kaplan-Meier curve demonstrated that the overall survival of patients in the High-Risk group was significantly lower than that in the Low-Risk group (Figure 7C).

**Figure 8:**
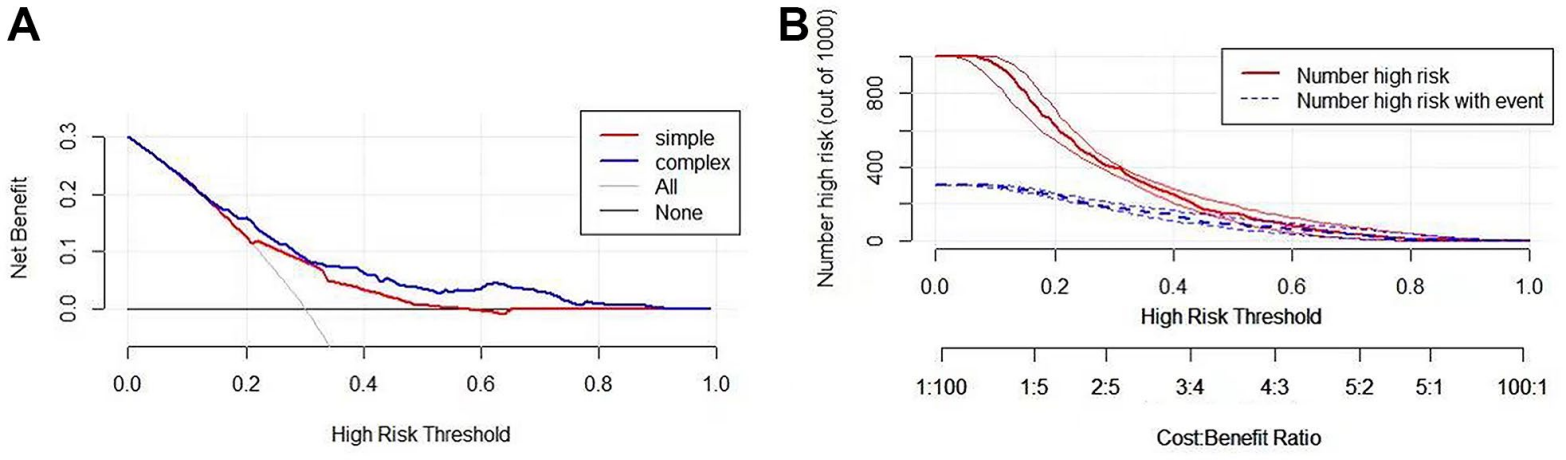
**Validation of the clinical value of the prognostic model.** (**A**) Drawing a decision analysis curve: in the Pt range of about 0.1-0.9, this prognostic model has a better clinical application value than A single indicator. (**B**) Developing clinical impact curves to predict risk stratification of LUAD patients in 1000 persons using simple and complex models.

**Figure 9:**
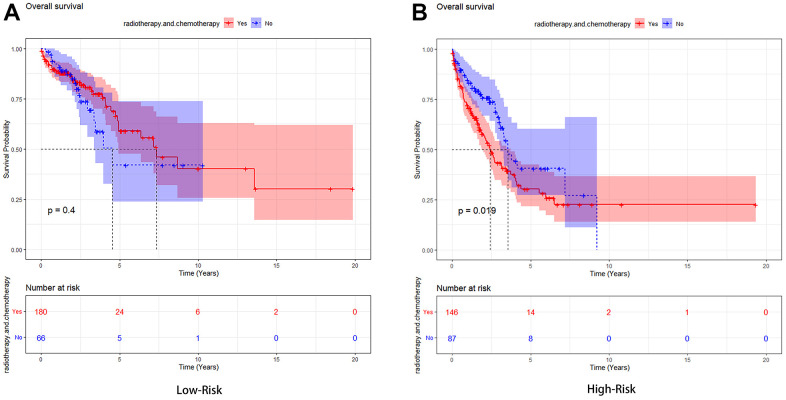
**Kaplan-Meier analysis of the high-risk group and low-risk group based on whether they had undergone radiotherapy and chemotherapy.** (**A**) The Low-Risk group’s Kaplan-Meier curve. (**B**) The High-Risk group’s Kaplan-Meier curve.

The area under the curve (AUC) values for predicting the three-year, five-year, and ten-year overall survival rate were 0.699, 0.673, and 0.727, respectively, demonstrating that this prognostic model had better sensitivity and specificity than other single indicators (Figure 7D). In addition, to increase the robustness of the clinical value of the prognostic model, decision curve analysis (DCA) was conducted. The results showed that: in the range of Pt of about 0.1-0.9, this prognostic model had a better clinical application value than a single indicator (Figure 8A). The clinical impact curve was further drawn, and the risk stratification in LUAD patients was predicted by a simple model and a complex model of 1000 persons, as shown in Figure 8B. Nevertheless, we further analyzed the Kaplan-Meier cumulative curves of the High-Risk and Low-Risk groups based on whether they had undergone radiotherapy and chemotherapy (excluding patients with no information on radiotherapy and chemotherapy recorded). The results showed no significant difference in OS of the Low-Risk group (*P*=0.4, Figure 9A). However, a significant difference in OS of the High-Risk group was found (*P*<0.05, Figure 9B).

### GSEA of LUAD patients with high-risk and low-risk characteristics

**Figure 10:**
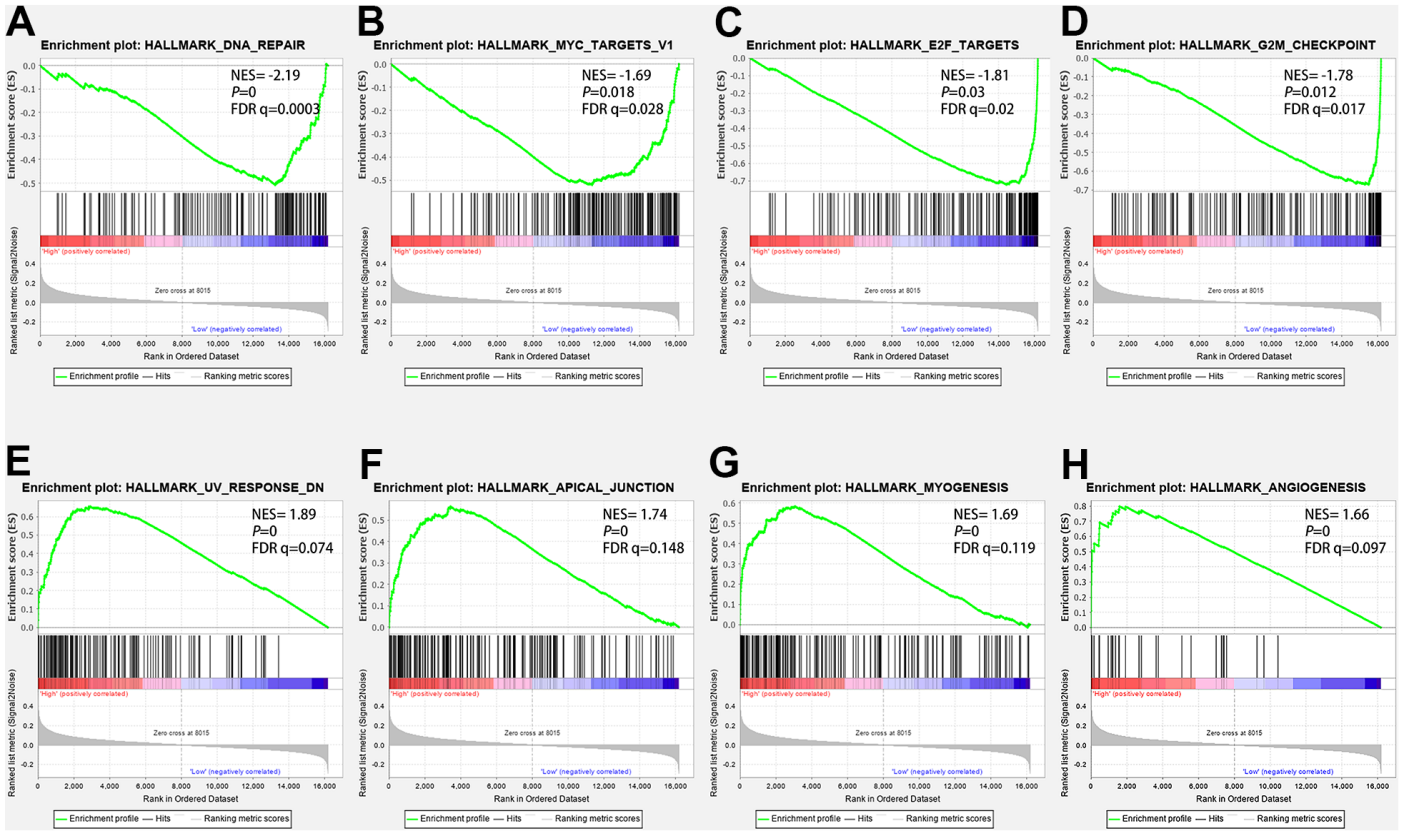
**The GSEA analysis results in the TCGA-LUAD cohort.** (**A**) “DNA repair”, (**B**) “Myc targets V1”, (**C**) “E2F target genes”, (**D**) “G2-M cell-cycle checkpoint”, (**E**) “UV response dn”, (**F**) “Apical junction”, (**G**) “Myogenesis”, (**H**) “angiogenesis”.

We performed GSEA to analyze related biological processes and signaling pathways associated with autophagy genes in high- and low-risk populations. We compared the gene expression profiles of high-and low-risk LUAD patients according to the characteristics of two autophagy-related genes in the training set. The GSEA results showed that genes in the high-risk group were mainly involved in the tumorigenesis pathway and myogenesis process, including UV response dn (NES= 1.89, *P*=0, FDR q=0.074), the apical junction (NES=1.74 *P*=0, FDR q=0.148), myogenesis (NES=1.69, *P*=0, FDR q=0.119), angiogenesis (NES=1.66, *P*=0, FDR q=0.097). Genes in the low-risk group were mainly involved in DNA repair (NES=-2.19, *P*=0, FDR q =0.0003), Myc targets V1(NES=-1.81, *P*=0.03, FDR q=0.02), E2F target genes (NES=-1.78, *P*=0.012, FDR q=0.017), G2M cell-cycle checkpoint (NES=-1.69, *P*=0.018, FDR q=0.028). The GSEA results are shown in Figure 10.

### Establishment of a nomogram model for LUAD patients

**Figure 11:**
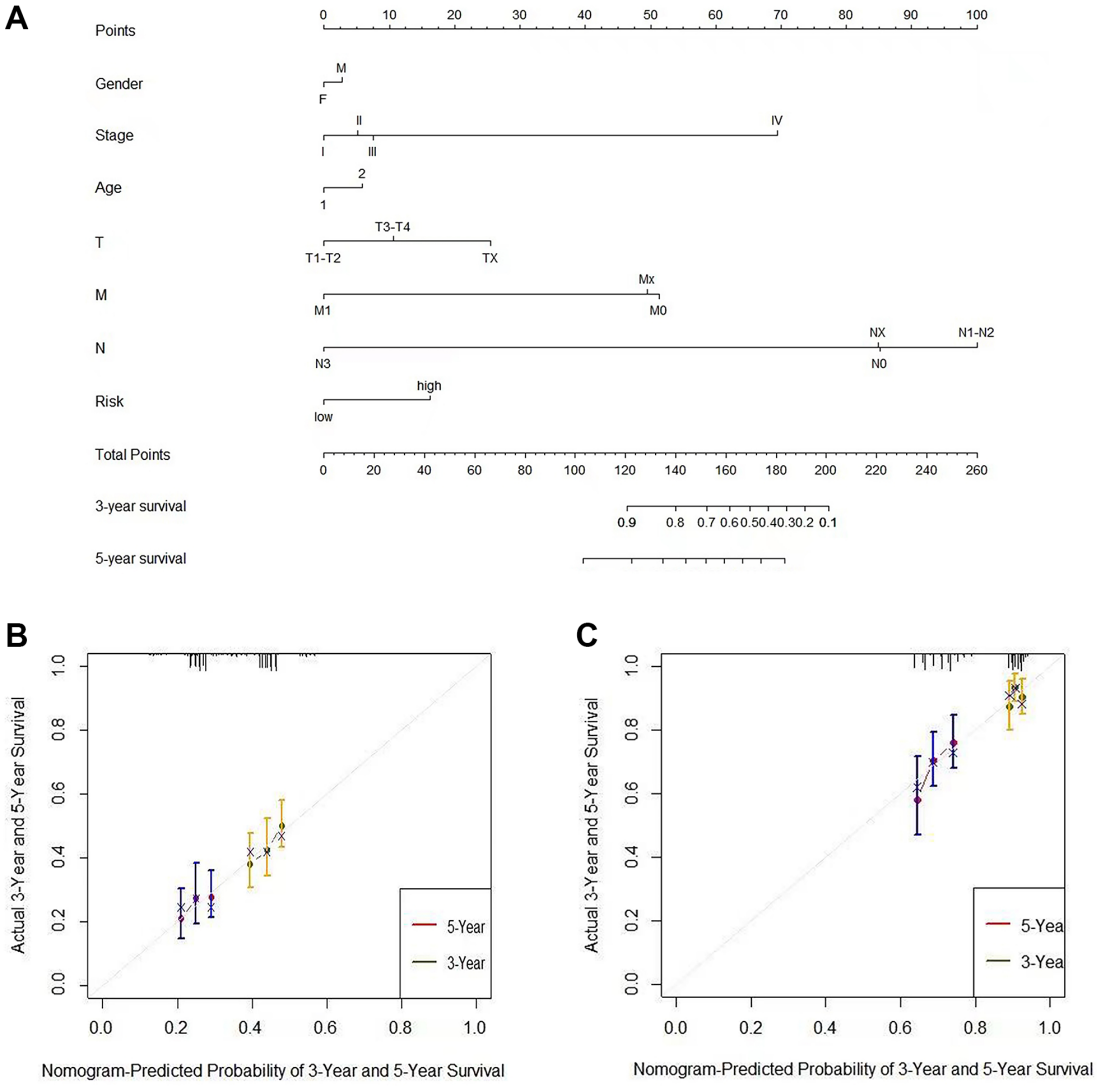
**The nomogram can predict the prognosis probability in LUAD.** (**A**) A nomogram of the TCGA-LUAD cohort (training set) was used to predict the OS. (**B**) Calibration maps were used to predict the 3-year and 5-year survival in the training set. (**C**) Calibration plots for 3-year and 5-year survival in the GSE68465 lung adenocarcinoma cohort (test group). The x-axis and y-axis represented the predicted and actual survival rates of the nomogram, respectively. The solid line represents the predicted nomogram, and the vertical line represents the 95% confidence interval.

A nomogram is a tool for predicting clinical outcomes with multiple risk factors based on multivariate regression analysis. In the present study, age, gender, and TNM staging were used to build a nomogram model to predict the three-year and five-year overall survival of the TCGA-LUAD cohort. As shown in Figure 11A, six corresponding lines were drawn for each patient according to the above six influencing factors, and the corresponding points were found on the total score axis to determine the score, and the C-index to evaluate the OS of the model was 0.738. After these scores were summarized, the probability of survival of the patients for three years and five years was determined by drawing lines downward from the total score axis. In addition, we obtained good consistency with the three-year and five-year calibration curves of the TCGA-LUAD cohort (Figure 11B) and validation dataset GSE68465 (Figure 11C).

### Protein expression patterns of risk genes

**Figure 12:**
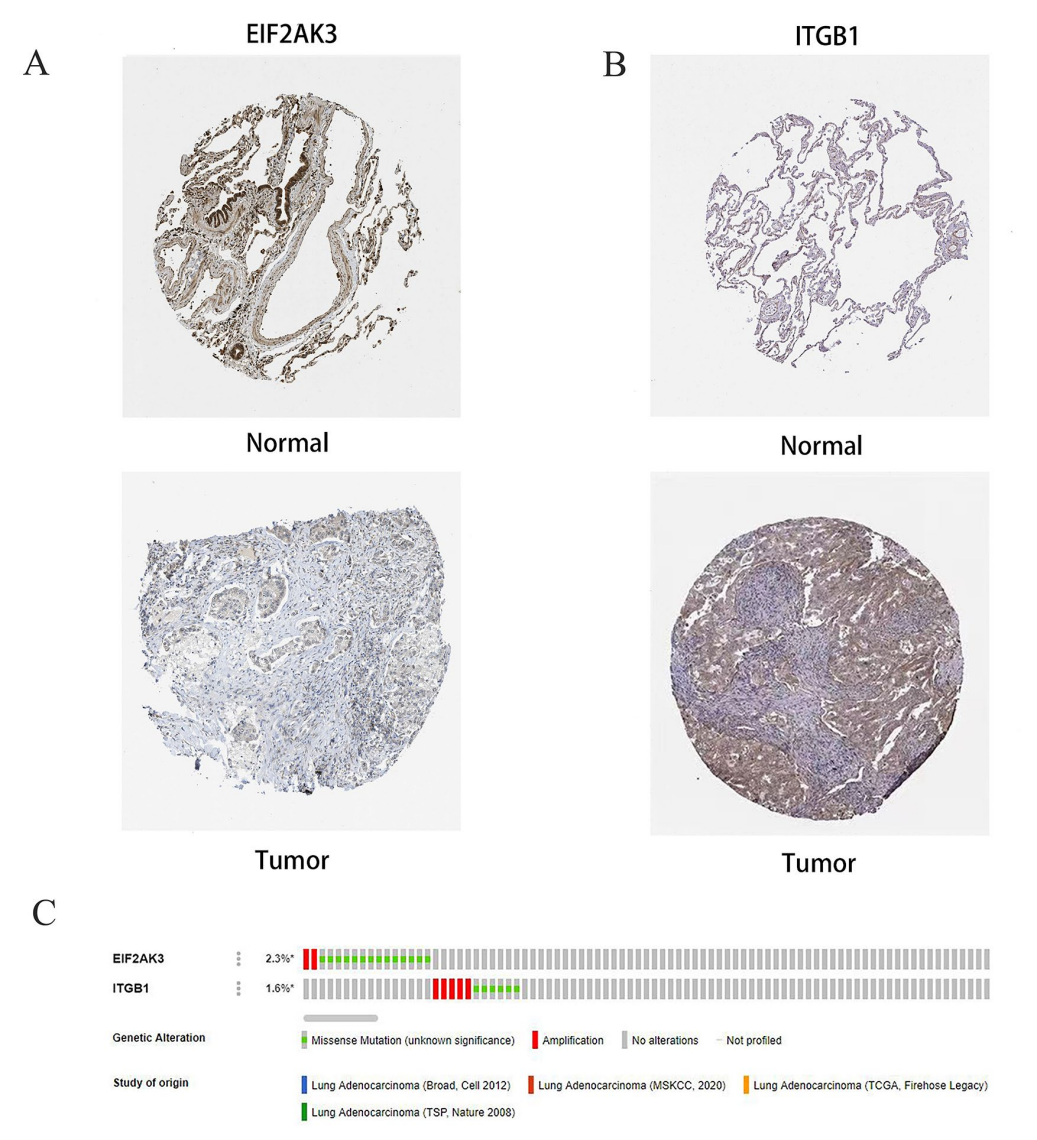
**Immunohistochemical (IHC) results and the copy number and mRNA expression levels of two ARGs in the autophagy prognostic model.** (**A**, **B**) The protein expressions of EIF2AK3 and ITGB1 were detected by the immunohistochemical method through the HPA database, and the staining intensity was labeled as low, moderate, and high. (**C**) OncoPrint showed the change of copy number and mRNA expression of two ARGs in the autophagy prognostic model.

We obtained the protein expression pattern of EIF2AK3 and ITGB1 genes in the HPA database to further verify their gene expression in the risk model (Figure 12). The results showed that EIF2AK3 was expressed at low levels in LUAD tissues and moderately expressed in normal lung tissues, ITGB1 was moderately expressed in normal lung tissues, and highly expressed in LUAD tissues. This observation was consistent with the mRNA expression levels of the genes we had previously observed. Furthermore, we analyzed the relationship between CNVs and mRNA expression levels of EIF2AK3 and ITGB1 genes through the cBioportal database. CNVs were found to be correlated with the mRNA expression of these genes (Figure 12C).

### RT-qPCR

**Figure 13:**
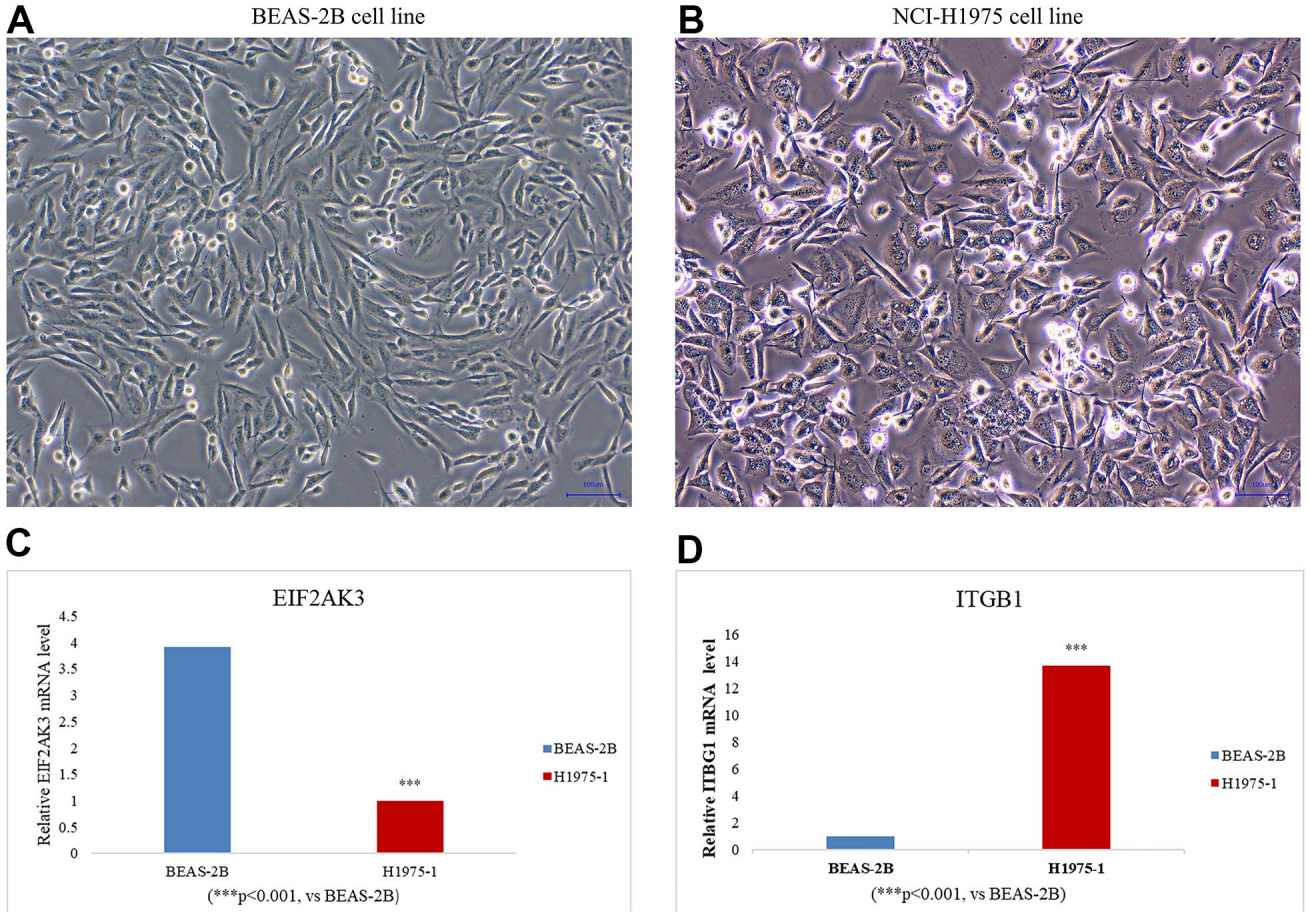
Cell line culture of (**A**) BEAS-2B (human normal lung epithelial cells) and (**B**) NCI-H1975 (human lung adenocarcinoma cells). The expression levels of EIF2AK3 (**C**) and ITGB1 (**D**) in LUAD cell lines were detected by RT -qPCR. LUAD cell line: NCI-H1975. Data were means ± SEM. ***P < 0.001. Experiments were repeated three times.

Human EIF2AK3 and ITGB-1 specific primers were designed and total RNA was extracted by NCI-H1975 (human lung adenocarcinoma cell) and BEAS-2B (human normal lung epithelial cell). Then, mDNA was used as a template to transcribe cDNA using random primers (HiScript III 1st Strand cDNA Synthesis Kit (+gDNA WIper), Nanjing, China). Then, the expression of the target gene was quantified using a qPCR fluorescence kit (SYBR GREEN qPCR MIXT Beijing, China). The cycle parameters were polymerase activation at 95° C for 30 seconds, followed by 40 cycles at 95° C for 5 seconds and 60° C for 30 seconds. GAPDH was used as an internal control. The expression of 2^-ΔΔCt^ was multiple changes in gene expression between the experimental group the and control group. All primer sequences are listed in Table 3. Meanwhile remarkably elevated levels of ITGB1 and demoted levels of EIF2AK3 were also verified by RT-qPCR in LUAD cell lines (Figure 13, Supplementary Material 4 - RT-qPCR Data).

**Table 3:** Primers and their sequences for RT-qPCR analysis.

**Primer**	**Sequence**
H-EIF2AK3-F	AAGGTTGGAGACTTTGGGTTAG
H-EIF2AK3-R	GAATCTGCTCTGGGCTCATATAC
H-ITGB1-F	CATGTTGTGGAGAATCCAGAGT
H-ITGB1-R	GCAGTAATGCAAGGCCAATAAG

## DISCUSSION

Worldwide, lung cancer is one of the most common cancers with high morbidity and mortality, irrespective of gender, accounting for about 2.1 million annual deaths and nearly one-fifth of the global cancer mortality rate [1]. Lung adenocarcinoma accounts for about 40% of all lung tumors [19]. Unfortunately, LUAD is a highly heterogeneous and invasive disease with overall survival of fewer than five years and is often associated with genetic alterations [3, 21]. Nowadays, with the rapid rise of next-generation sequencing technology and bioinformatics, more and more studies have begun to focus on the critical role of genes in predicting LUAD [22–24].

It is widely acknowledged that autophagy, as a process of self-degradation, plays the role of housekeeper genes in the clearance of intracellular pathogens and damaged organelles [8]. Autophagy can be divided into macroautophagy (MA), microautophagy (MI), or chain-mediated autophagy (CMA) [23]. Autophagy deficiency has been associated with many diseases and tumor pathogenesis [24–28], where it plays a dual role in different cancer stages, acting as an inhibitor of tumor development in early stages and exhibiting a protective effect on cancer cells leading to invasion and metastasis in advanced stages [21, 29].

Autophagy is closely bound to the occurrence, development, and prognosis of tumors [30, 31]. Many studies have assessed the correlation between cancer prognosis and autophagy and the construction of a prognosis model, such as screening autophagy-related clinical prognostic indicators and the construction of a clinical prognosis model in patients with gastric cancer, colorectal cancer, and thyroid cancer [32–35].

A study of 66 NSCLC patients found that patients with high LC3 expression had a better prognosis than those with low LC3 expression [36]. Furthermore, a systematic review and meta-study of p62/SQSTM1 in lung cancer patients found that overexpression of p62 was linked to overall survival in lung cancer patients [37, 38]. Beclin-1 expression was negatively correlated with tumor size and tumor stage of lung adenocarcinoma, and Beclin-1 expression was decreased in NSCLC tissues compared with normal tissues [39]. Considering that autophagy plays a vital role in cancer development and existing studies on the correlation between autophagy genes and adenocarcinoma [40, 41], we hypothesized that ARGs would have a great prospect in the prognosis assessment of lung adenocarcinoma. A prognosis model obtained by combining various algorithms would play a role in predicting overall survival in LUAD patients.

Herein, we sought to establish a model consisting of autophagy-related genes, which can accurately predict of prognosis of LUAD patients. We analyzed the expression of 210 ARG genes from the TCGA-LUAD dataset. GO, and KEGG pathway enrichment analysis results confirmed involvement in the autophagy process in LUAD. The univariate Cox proportional hazards model showed that 27 ARGs were associated with overall survival. We further developed 13 prognostic markers from the TCGA-LUAD cohort by LASSO regression and characterized two autophagy genes (ITGB1 and EIF2AK3) by the multivariate Cox risk regression model. In addition, the protein expression and CNVs of the above two genes were validated in the HPA (Human Protein Atlas) database and the Cbioportal database.

Moreover, we calculated the risk score for each LUAD patient using the mRNA expression level of the selected genes and the corresponding risk factor. In the TCGA-LUAD cohort, patients were stratified based on the risk score. More importantly, we used the GEO dataset GSE68465 to further validate the above results. In addition, our GSEA results showed that pathways such as cell cycle and DNA repair were enriched in the Low-Risk group, while the High-Risk group was involved in tumor progression and exhibited significant differences in signaling pathways.

Integrin-β (ITGB) superfamily is one of the Integrin superfamily, which consists of 8 different members in the human body [42]. ITGB1 is a subunit of the isomer transmembrane receptor, formed by binding to the ITGA subunit or corresponding ligand, and is associated with FAK phosphorylation at tyrosine 397. Based on these advances, more and more studies have shown that ITGB1 has the potential to regulate cell-matrix interaction, cell proliferation, diffusion, metastasis, and even the progression of EMT (epithelial-mesenchymal transformation) [43]. Previous studies have confirmed that integrin β1 deficiency increases myocardial dysfunction and apoptosis after myocardial infarction [44]. Previous studies have shown that integrin β1 (ITGB1) is overexpressed in tumor cells and is involved in angiogenesis, tumor progression, and metastasis [45]. Interestingly, meta-studies found that the high-level expression of ITGB1 was significantly correlated with the overall survival difference in lung and breast cancer patients. No correlation was found between the high expression of ITGB1 and overall survival in colorectal cancer [46]. Moreover, high expression of ITGB1 has been reported to significantly promote the invasion of gastric cancer [45]. Compared with TNM staging, ITGB1 overexpression has been reported to predict a poor prognosis of pancreatic cancer [47]. In addition, the upregulated expression of ITGB1 was also significantly correlated with tumor metastasis and tumor necrosis [48]. An increasing body of evidence suggests that ITGB1 is abnormally highly expressed in various solid cancers, and ITGB1-DT promotes the development of LUAD by forming a positive feedback loop with ITGB1/Wnt/β-catenin/Myc [49].

PERK (also known as PEK, EIF2AK3) is a type I transmembrane protein with a serine/threonine cytoplasmic domain [50]. It is activated primarily by the accumulation of misfolded proteins in the endoplasmic reticulum (ER) [51]. The role of PeRK on a physiological level is unclear. It has been established that the loss of PERK is the cause of Human Wolcott -- Rallison syndrome (wRS) [52]. Another physiological function of PeRK appears to be to promote mammary maturation [53]. ER stress has been implicated in the pathophysiology of many diseases, including heart disease, cancer, and neurodegenerative diseases [54]. Secreted proteins and membrane protein synthesis in ER are essential for the normal contractile function of cardiomyocytes. Inadequate adaptive ER-PQC (endoplasmic reticulum protein quality control) and UPR (unfolded protein response) in cardiomyocytes lead to protein toxicity, accumulation of end-misfolded proteins, aggregation formation, and ultimately damage to cardiomyocyte contractile function and heart failure [55]. The relationship between PERK and cancer progression is a particularly interesting aspect of the PERK signal. It is a well-established fact that solid tumors are prone to hypoxic areas, and hypoxic tumors are particularly aggressive and chemically resistant Hypoxia is a potent inducer of PERK dependent eIF2α phosphorylation, consistent with PERK’s important pro-survival function in hypoxia-exposed cells, where PERK signaling increases tumor size, vascularization, and cell survival [53]. Early studies have shown that PERK (EIF2AK3) plays a role in tumor angiogenesis [56]. Dai et al. [57] have shown that activating the PERK/eIF2/ATF4 signaling pathway in pancreatic adenocarcinoma cells can prevent tumor progression. Glowi et al. regulated the drug resistance and clonal survival of cancer through mutual regulation between GCN2 (EIF2AK4) and PERK (EIF2AK3) [58]. In breast cancer, the expression of PERK (EIF2AK3) was down-regulated, but its activity was shown to be constitutionally elevated in drug-resistant cells, and it was found that PERK could be a potential target for drug-resistant cancer therapy [58]. Wang et al. found that silica nanoparticles (SINPs) induced the accumulation of autophagosomes by activating the EIF2AK3 and AtF6 UPR pathways in liver cells [59].

However, to the best of our knowledge, no studies on autophagy-related genes combined with clinicopathological parameters to predict LUAD have been reported. In this study, using the TCGA-LUAD dataset, we found 31 ARGs mainly related to neuronal death, glutamate receptor signaling pathway, neuronal apoptosis signaling pathway, autophagy regulation, and endopeptidase activity. Glutamate is a signaling medium that stimulates the proliferation of non-neuronal tumor cells [60, 61]. It is worth noting that many studies have found that tumor cells from neuronal tissue express the Ionic Glutamate Receptor (IGLUR) subunit, which is differentially expressed in a variety of cancer cells, such as lung cancer, thyroid cancer, breast cancer, gastric cancer, and glioblastoma multiforme [62, 63]. Moreover, Xiao et al. found that the upregulated expression of glutamate receptor GRM4 can observably inhibit the proliferation of breast cancer cells and reduce the migration and invasion ability [64].

In our constructed LUAD prognosis model, we found ARGs ITGB1 and EIF2AK3 played opposing roles as “risk” and “protective” genes in the prognosis of LUAD. In addition, we conducted a risk score and survival analysis, which demonstrated that the High-Risk group had a significantly poorer prognosis than the Low-Risk group. This suggests that our prognostic model can be used to predict the prognosis of LUAD patients. Furthermore, we combined autophagy-related genes with clinicopathological parameters of the TCGA-LUAD cohort to plot the three-year and five-year survival rates. Calibration curves showed that the predicted survival rates of LUAD patients were consistent with the actual survival rates, which indicated that the constructed ARG-based LUAD prognostic model has diagnostic value in predicting prognosis.

Based on this, we believe that the model constructed by these two autophagy genes and clinicopathological parameters allows for more accurate prognoses for patients with lung adenocarcinoma. We concede that ITGB1 and EIF2AK3 have limitations in predicting the prognosis of lung adenocarcinoma. Our study only tested and validated our findings in two databases. Meanwhile, in the LUAD cell line, it was also confirmed by RT-qPCR that the ITGB1 level was significantly increased and the EIF2AK3 level was significantly decreased (Figure 13). Indeed, a robust and reliable prediction model that can be implemented at the clinical level requires further validation in a multicenter study with larger sample size. Furthermore, functional experiments *in vitro* are essential to further explore the possible mechanisms underlying the role of autophagy genes in LUAD.

## CONCLUSIONS

In conclusion, our study established a novel model based on two autophagy-related gene signatures and a nomogram based on the mRNA expression levels in LUAD patients to predict the prognosis of lung adenocarcinoma patients, which will provide valuable information for the diagnosis of LUAD patients and the development of new treatment modalities.

## MATERIALS AND METHODS

### Data mining

We downloaded the gene expression profiles of LUAD with corresponding clinical data of patients from the Cancer Genome Atlas (TCGA; https://portal.gdc.cancer.gov/) and the Gene Expression Omnibus (GEO; https://www.ncbi.nlm.nih.gov/geo/) database. There were 535 LUAD and 59 non-tumor cases of the RNA-seq expression data from the TCGA-LUAD cohort (Supplementary Material 2 - TCGA-LUAD Clinical Data). Moreover, we also searched the GEO database to download RNA-Seq data by setting a filter: (1) Case Number: at least 100 cases; (2) Type: expression profiling data; (3) Including survival data. Ultimately, 439 patients who had complete survival information obtained from the GSE68465 dataset were included in our study (Supplementary Material 3 - GEO68465 Clinical Data). The Human Autophagy-dedicated Database (HADb; http://www.autophagy.lu/autophagy.html) was used to search for autophagy-related genes (Supplementary Material 1 - Autophagy genes).

### Differential expression, enrichment analysis, and PPI networks of ARGs in LUAD

The r package “limma” (version 4.0.5) was used to perform the DEG analysis. The screening criteria for DEGs were |Log2FC|>1 and an adjusted *P*-value<0.05. Gene Ontology (GO) and the Kyoto Encyclopedia of Genes and Genomes (KEGG) enrichment analyses were carried out with the R package “clusterprofileiler”. The STRING database (https://string-db.org/) was used to build a protein-protein interaction (PPI) network of autophagy-related genes.

### Prognostic model establishment and validation

Potential ARGs were screened out using the univariate Cox proportional hazards model according to the overall survival of LUAD patients (*p*<0.01). To avoid overfitting and reduce the number of prognostic predictors used to predict overall survival, the LASSO Cox regression analysis was utilized by the R package “glmnet” to screen the genes. Next, we used the multivariable Cox proportional hazards model to construct the prognosis model of LUAD-ARGs. We calculated the risk score by adding the expression value of each factor×the value of the regression coefficient. Then the optimal cutoff values for the risk score were calculated (utilizing the R packages “survival”, “survminer”, and “bilateral test”). Finally, the LUAD patients were divided into Low-Risk and High-Risk groups according to the cutoff value.

### Gene set enrichment analysis

GSEA (version 4.1.0, http://www.broadinstitute.org/gsea/index.jsp) was used to assess biological pathways or gene sets that differ significantly between the High-Risk and Low-Risk groups. Parameters were set as follows: | NES | > 1, *P*< 0.05, FDR q < 0.25.

### The establishment of the prognostic nomogram

Age, Gender, Risk score, and other clinicopathological characteristics were chosen to establish the nomogram in the company by using the R packages “rms” and “survival”. Then, the consistency between actual and predicted survival through calibration curves was assessed.

### Analysis of these vital ARGs expression level

To further confirm results from the above analysis, the Human Protein Atlas (HPA) database (http://www.proteinatlas.org/) was wielded to identify the expression of these ARGs at the protein level in LUAD tissues and normal tissues. cBioPortal (https://www.cbioportal.org/), a visual analytic tool, was used to analyze the ARGs expression.

### Statistical analysis

The Kaplan-Meier Plotter was used to analyze the difference in survival time between the two different risk groups, using the R package “timeROC” to plot ROC curves and assess the Risk Score’s sensitivity and specificity for prognosis prediction. A time-dependent ROC curve was performed to compare the accuracy of three-year, five-year, and ten-year overall survival predictions. We used Decision Curve Analysis to evaluate the clinical prediction model as a supplement to ROC curve analysis to further confirm the feasibility of the prognostic model.

### Availability of data and materials

We obtained datasets from publicly available databases for this analysis. These data are available in the following databases: TCGA: https://portal.gdc.cancer.gov/; GEO: https://www.ncbi.nlm.nih.gov/geo/; HADb: http://www.autophagy.lu/autophagy.html.

In addition to the gene expression data of LUAD downloaded from the TCGA database, the original data in this manuscript also included clinical data of TCGA-LUAD, and clinical data of the GSE68465 dataset in the GEO database, collation of autophagy-related genes, and RT-qPCR data of EIF2AK3 and ITGB1. They can be found in the four documents of TCGA-LUAD Clinical Data, GEO68465 Clinical Data, Autophagy Genes, and RT-qPCR Data in the Supplementary Materials.

## Supplementary Material

Supplementary Material 1

Supplementary Material 2

Supplementary Material 3

Supplementary Material 4
